# Identifying Effective Feature Selection Methods for Alzheimer’s Disease Biomarker Gene Detection Using Machine Learning

**DOI:** 10.3390/diagnostics13101771

**Published:** 2023-05-17

**Authors:** Hala Alshamlan, Samar Omar, Rehab Aljurayyad, Reham Alabduljabbar

**Affiliations:** Department of Information Technology, College of Computer and Information Sciences, King Saud University, P.O. Box 145111, Riyadh 4545, Saudi Arabia; 438204382@student.ksu.edu.sa (S.O.); 438203529@student.ksu.edu.sa (R.A.);

**Keywords:** data mining, genetic disease prediction, Alzheimer disease, gene expression, feature selection, classification

## Abstract

Alzheimer’s disease (AD) is a complex genetic disorder that affects the brain and has been the focus of many bioinformatics research studies. The primary objective of these studies is to identify and classify genes involved in the progression of AD and to explore the function of these risk genes in the disease process. The aim of this research is to identify the most effective model for detecting biomarker genes associated with AD using several feature selection methods. We compared the efficiency of feature selection methods with an SVM classifier, including mRMR, CFS, the Chi-Square Test, F-score, and GA. We calculated the accuracy of the SVM classifier using validation methods such as 10-fold cross-validation. We applied these feature selection methods with SVM to a benchmark AD gene expression dataset consisting of 696 samples and 200 genes. The results indicate that the mRMR and F-score feature selection methods with SVM classifier achieved a high accuracy of around 84%, with a number of genes between 20 and 40. Furthermore, the mRMR and F-score feature selection methods with SVM classifier outperformed the GA, Chi-Square Test, and CFS methods. Overall, these findings suggest that the mRMR and F-score feature selection methods with SVM classifier are effective in identifying biomarker genes related to AD and could potentially lead to more accurate diagnosis and treatment of the disease.

## 1. Introduction

Alzheimer’s disease (AD) is a prevalent form of dementia that accounts for up to 80% of all dementia cases. It is characterized by a progressive and irreversible decline in brain function, resulting in the loss of neurons, memory, and cognitive abilities. Unfortunately, there is currently no cure for AD, and it disproportionately affects elderly individuals [1,2]. In the early stages of the disease, memory loss is mild, but as the disease progresses, patients may lose the ability to carry on conversations. Dementia, including AD, is a global health concern, with approximately 50 million people worldwide diagnosed with the disease in 2018 [3]. Furthermore, the annual number of new cases of AD and other forms of dementia is projected to triple by 2050, with one new case of dementia diagnosed every three seconds [3]. While researchers believe that there is no single cause of AD, multiple factors such as genetics, age, lifestyle, and environment may contribute to its development [4].

Genetic inheritance is responsible for up to 5% of AD cases, with mutations in genes such as APP, APOE, PSEN1, and PSEN2 linked to the disease between 1987 and 1993 [4,5]. Gene expression refers to the process by which genes are transcribed into messenger RNA (mRNA), which then directs the synthesis of proteins that perform various functions within cells. Changes in gene expression can be indicative of alterations in cellular processes and signaling pathways, which may contribute to the development of diseases such as Alzheimer’s disease (AD) [2]. In recent years, there has been increasing interest in using gene expression data to predict the onset of AD. This involves analyzing the expression levels of different genes in biological samples, such as blood or cerebrospinal fluid, from individuals with or without AD. By comparing the gene expression patterns between the two groups, researchers can identify genes that are differentially expressed, i.e., those that are more highly expressed in one group than the other.

The use of machine learning and feature selection methods in predicting AD based on gene expression data is a rapidly evolving area of research. As more datasets become available, researchers can evaluate the effectiveness of different models and feature selection methods in predicting AD. Additionally, future research could explore the potential of hybrid approaches that combine filter and wrapper models for feature selection in AD prediction. Using machine learning algorithms, researchers can then develop a predictive model that incorporates these differentially expressed genes as features. The model is trained on a set of samples with known disease status (AD or control) and then tested on a separate set of samples to assess its accuracy in predicting disease status. Several studies have reported promising results using gene expression data to predict AD. For example, a recent study published in the journal *Frontiers in Aging Neuroscience* used a machine learning approach to identify a set of 13 genes that could accurately distinguish between AD patients and healthy controls with an accuracy of 93.1% [1]. Another study published in the journal *Neurobiology of Aging* identified a set of 17 genes that were differentially expressed in AD patients and could predict disease status with an accuracy of 87% [2].

Overall, gene expression analysis holds great promise for improving the early diagnosis and prediction of AD. However, further research is needed to validate these findings and develop more robust predictive models that can be used in clinical practice. Early and accurate diagnosis of AD plays a crucial role in prevention, treatment, and patient care, particularly in the early stages of the disease. Currently, the diagnosis of AD is primarily performed through analysis of brain images such as MRI and PET. However, it is also important to identify which genes are altered in AD patients, especially in the early stages of the disease, to better control its progression. Recent studies in the fields of Bioinformatics and Biomedicine have employed various statistical, machine learning, and data mining techniques to identify useful data patterns for detecting several diseases [6]. This study aims to develop an intelligent data mining model for the prediction of AD based on gene expression.

The paper is organized as follows. In the next section, we provide a brief background on Alzheimer’s disease (AD), machine learning techniques, and feature selection methods. Section 3 provides an overview of previous research on genetic disease prediction. In Section 4, we describe the dataset used in this study and the proposed model architecture. Section 5 outlines the experimental setup and presents the results. In Section 6, we discuss our method and the findings in detail. Section 7 presents our plans for future work and concludes the study’s output.

## 2. Background

In this section, we provide a brief background on Alzheimer’s disease (AD). We also introduce machine learning techniques, including supervised machine learning, and feature selection methods. By understanding these concepts, we can better appreciate the importance of machine learning and feature selection in predicting and diagnosing AD.

### 2.1. Alzheimer’s Disease

Alzheimer’s disease (AD) is a progressive brain disorder that was first described by Dr. Alois Alzheimer in 1906. Dr. Alzheimer observed symptoms in his patient, such as memory loss, paranoia, and psychological changes, and upon autopsy, he noticed shrinkage of the patient’s brain [7]. AD is the most common cause of dementia, which is a condition that slowly destroys memory and cognitive functioning, ultimately impacting the ability to carry out daily activities [8].

Currently, AD is ranked as the sixth leading cause of death worldwide, and the symptoms typically appear in individuals over the age of 60 [8]. In Saudi Arabia, experts estimate that 3.23% of the population, mostly aged 65 or older, may have dementia caused by AD [9]. Despite extensive research efforts, there is currently no cure or definitive treatment for AD. Current approaches to managing the disease focus on helping individuals maintain cognitive function, manage behavioral symptoms, and slow the progression of memory loss [8]. However, researchers are actively pursuing therapies that target specific genetic, molecular, and cellular mechanisms in the hopes of stopping or preventing the underlying cause of the disease [8]. The complex nature of AD makes it a challenging condition to treat and manage. However, continued research and innovative approaches may lead to more effective treatments and improved outcomes for individuals with AD and their families.

### 2.2. Supervised Machine Learning

Supervised Machine Learning (SML) is when a machine is programmed to find particular patterns in massive data. SML has different ways to adjust these data by adjusting the algorithm to make predictions and many other tasks [10]. The term is directly related to the fields of programming, IT, and mathematics. It is applied in all types of sectors of government, marketing, medicine, and any business which collects data and wants to make a decision based on these data. Subsequently, it is employed in consumer choices, weather forecasting, and website calculations. In this paper, we concentrate on various types of SML [10]. Although there are many, many categories and aspects to SML, we would only generally describe the following: Support Vector Machine, Logistic Regression, Linear Discriminant Analysis, K-nearest neighbor, Decision Tree, and Naïve Bayes.

#### Support Vector Machine (SVM)

SVM is a discriminative classifier formally defined by a separating hyperplane. The algorithm outputs an optimal hyperplane which categorizes new examples. In two-dimensional space, this hyperplane is a line dividing a plane into two parts. Each class is separated on each side of the plane [10]. A hyperplane is a line that linearly separates and classifies a set of data. Generally, the further from the hyperplane our data points lie, the more confident we are that they have been correctly classified. Hence, when new testing data are added, whatever side of the hyperplane they land on will decide the class that we assign to them [10].

Given the solutions ${\hat{\beta }}_{0}$ and $\hat{\beta }$, the decision function can be written as

(1) $$\hat{G}\left(x\right)=\mathrm{sign}\left[\hat{f}\left(x\right)\right]=\mathrm{sign}\left[x^{T}\beta +{\hat{\beta }}_{0}\right]$$

One aspect of SVM is its accuracy. SVM works well on smaller cleaner datasets. It can be more efficient because it uses a subset of training points. The cons are that it is not suited to larger datasets, as the training time with SVM can be high [10].

### 2.3. Feature Selection

Feature selection is a process of removing irrelevant features in a dataset, where the chosen algorithm automatically selects those features that contribute most to the prediction variable or output in which one is interested. Using feature selection before fitting data into the classifier can enhance accuracy by reducing training time and overfitting.

#### 2.3.1. mRMR

This stands for minimum Redundancy Maximum Relevance. mRMR aims to select the genes that have shown low correlation among the other genes (Minimum Redundancy) but still have high correlation to the classification variable (Maximum Relevance). For classes *c* = (ci, … ck) the Maximum Relevance condition is to maximize the total relevance of all features in

(2) $${max}_{S\subset \Omega }\frac{1}{\left|S\right|}\sum_{i\in S}I(c,f_{i})$$

The Minimum Redundancy condition is

(3) $${min}_{S\subset \Omega }\frac{1}{{\left|S\right|}^{2}}\sum_{i,j\in S}I(f_{i},f_{j})$$

where $f_{i}$ and $f_{j}$ refer to the expression levels of genes *i* and *j*, respectively. 

#### 2.3.2. CFS

CFS stands for Correlation-based Feature Selection algorithm. CFS selects attributes by using a heuristic which measures the usefulness of individual genes for predicting the class label along with the level of inter-correlation among them. Highly correlated and irrelevant features are avoided. The method calculates the merit of a subset of *k* features as:

(4) $${Merit}_{S_{k}}=\frac{{kr}_{cf}}{\sqrt{k+k(k-1)\bar{r_{ff}}}}$$

Here, $\bar{r_{cf}}$ is the average value of all feature–classification correlations, and $\bar{r_{ff}}$ is the average value of all feature–feature correlations. The CFS criterion is defined as follows:

(5) $$CFS={max}_{S_{k}}\left[\frac{r_{cf1}+r_{cf2}+\cdots +r_{cfk}}{\sqrt{k+2\left(r_{f1f2}+\cdots +r_{fifj}+\cdots +r_{fkf1}\right)}}\right]$$

#### 2.3.3. Chi-Square Test

The Chi-Square Test is a statistical algorithm used by classification methods to check the correlation between two variables. In the following equation, high scores on χ_2_ indicate that the null hypothesis (H0) of independence should be eliminated and thus that the occurrence of the term and class are dependent:

(6) $$X^{2}=\sum \frac{{\left(observed-expected\right)}^{2}}{expected}$$

#### 2.3.4. F-Score

F-score is a simple statistical algorithm for feature selection. F-score can be used to measure the discrimination of two sets of real numbers.

#### 2.3.5. GA

Genetic Algorithm (GA) is one of the common wrapper gene selection methods. It is usually applied to discrete optimization problems. The main goal of GA is discovering the best and perfect solution within a group of potential solutions. This method reflects the process of natural selection where the fittest individuals are selected for reproduction in order to produce offspring of the next generation. Each set of solutions is named a population. Populations consist of vectors, i.e., chromosomes or individuals. Every item in the vector is referred to as a gene [11].

## 3. Related Works

This section will investigate different approaches used to detect AD, as well as highlight the derived benefits from previous work that used machine learning algorithms with genetics. From their experiments, 18 AD datasets have been investigated, In Table 1 we have listed all the datasets. All the works have been summarized in Table 2.

Paylakhi et al. [12] proposed an ADG identification approach, which is a novel hybrid method to identify relevant genes of AD. The proposed approach consists of three stages. In the first stage, they applied a Fisher criterion method as a filtering method to reduce noise and eliminate redundant genes from the dataset. Then the SAM technique was applied to identify genes with notable changes in their expression in order to increase the number of relevant genes. In the final stage, they used GA for optimization of the feature selection and SVM for classification. The proposed approach was tested on the AD GSE1297 dataset consisting of 31 samples provided by the Gene Expression Omnibus (GEO) database. The results show that the proposed method obtains 94.55% accuracy with 44 genes identified. Analysis of the 44 identified genes by GO and KEGG led to the identification of AD-related terms and pathways.

Voyle et al. [13] investigated the robustness of Pathway Level Analysis of Gene Expression (PLAGE) across gene expression samples from different populations. The study used a combined dataset which contains 748 subjects of peripheral-blood gene expression obtained from Add Neuro Med (ANM) and Dementia Case Registry (DCR) cohorts. Random Forest (FR) models were used with recursive feature elimination in particular to differentiate patients with AD or mild cognitive impairment (MCI) from healthy elderly controls (CTL). The authors state that there was no observable difference in the performance metrics of gene expression and PLAGE model.

Yu Miao et al. [14] proposed an ADG identification algorithm that combines feature selection, cascading classifier, and majority voting. This is in addition to multiple classifier integration such as SVM, RF, and extreme learning machine (ELM). In addition, a feature selection algorithm called ReliefF was applied to select the most relevant attributes and improve accuracy. The proposed method was tested on the AD dataset of 22,283 genes of 31 patients provided by The National Center for Biotechnology Information (NCBI). The results show that the proposed method obtains 78.77% sensitivity, 83.1% specificity, and 74.67% accuracy. Furthermore, based on the proposed method, they listed the top 13 genes predicted to be ADGs with a probability higher than 85%.

Another related work is found in the study elaborated by Martínez-Ballesteros et al. [15]. Their work was focused on analyzing the gene expression profiles related to AD using three integrated machine learning techniques (Decision Trees, Quantitative Rules, and Hierarchical Cluster). The purpose was to identify genes highly related to AD, through changes in their expression levels between control and AD samples. To fulfill this purpose, six phases were applied in a dataset consisting of 33 samples and 1663 genes provided by Dunckley et al. [16]. In the first three phases, the C4.5 algorithm with a minimum threshold for accuracy and the GarNet algorithm were applied to establish the best configuration settings for the model. In the fourth and fifth phases, gene groups were validated using hierarchical cluster analysis, including statistical tests and a biological knowledge integration process based on the information fusion obtained from a prior phase. Finally, they validated the results using additional data and a permutation test in the sixth phase. Their proposed method successfully characterized more than 90 genes whose expression is modified during AD progression.

On the other hand, Park et al. [17] concentrated on constructing AD disease-specific gene networks and disease mechanisms using SML techniques. The study presents a novel approach for gene–gene interaction (GGI) identification, where a Random Forest algorithm was applied over heterogeneous gene expression profiles to determine significant GGIs. They obtained a dataset consisting of 257 normal genes and 439 AD genes by selecting two associated gene expression profiles (GSE33000 and GSE44770). To define their features from an expression profile, they extracted 22 features including statistical measurements of gene expression and two correlation-based similarity measures, PCC and MI. In addition, to assign labels for the gene pairs, various interactome datasets and gene sets were utilized. To evaluate their proposed method, they compared the classification performance among various ML algorithms, Naïve Bayes, SVM, ANN, and PART, and found that their proposed model outperformed all the algorithms.

In addition to that, Huang et al. [18] published one of the Genome-Wide Association Studies (GWAS) on AD to identify disease-risk genes in the progress of disease and the complex networks of GGI. In their study a prediction algorithm for AD candidate genes was implemented based on an SVM classifier. A dataset consisting of 22,646 brain-specific gene expression networks was collected and then distributed among four datasets (AD-associated set, Control dataset, AD-predicted dataset, and Non-mental-health dataset). To find the significant differences among all four datasets, a set of features were extracted. Thereafter, specific coefficients were used to predict the level of potential AD association for every gene in the complex networks. As a result, the proposed algorithm classified AD candidate genes with an accuracy of 94% and an area under the Receiver Operating Characteristic (ROC) of 84.56%.

In 2019, Park et al. [19] published another study that proposed an AD prediction model based on a deep neural network using a multi-omics dataset. The authors integrated two heterogeneous omics datasets: large-scale gene expression and DNA methylation datasets. They obtained a large-scale gene expression dataset by integrating two gene profiles (GSE33000 and GSE44770) [20,21] to increase the sample size. The integrated dataset consisted of 257 normal control and 439 AD samples. In addition, a DNA methylation dataset consisting of 8 normal and 74 AD samples was provided by Smith et al. [22]. In this study, the authors applied a feature selection method that consisted of two steps to reduce the number of features. Firstly, they identified differentially expressed genes (DEG) and differentially methylated positions (DMP) and used the “Limma package” for analysis of both DEG and DMP. Second, they integrated DEGs and DMPs by intersecting. In addition, they developed a prediction model based on a Deep Neural Network (DNN) model and applied Bayesian optimization on the proposed model to investigate its optimized hyperparameters. The results of the proposed prediction model shows that it outperformed conventional machine learning algorithms such as Random Forest, SVM, and Naïve Bayesian. The highest accuracy of the conventional machine learning algorithm achieved was 0.7 for Random Forest, while the average accuracy of the proposed prediction model was 0.823.

**Table 1:** Alzheimer’s gene datasets list.

Ref.	Year	Dataset Name	Sample Size	Dataset Code
Blalock et al. [23]	2004	NCBI-GEO-GSE1297	31 samples, 22 AD (7 incipient, 8 moderate, and 7 severe), 9 NC, and 22,283 genes	D1
		NCBI-GEO-GSE63060	356 samples, 100 AD	D2
		NCBI-GEO-GSE63061	411 samples, 118 AD	D3
Dunckley et al. [16]	2006	GSE39420-GSE4757 GSE5281-GSE28146	33 samples, 20 AD, 13 NC, 35,722 probesets, and 1663 genes	D4
Narayanan et al. [20]	2014	NCBI-GEO-GSE33000	310 AD, 157 NC, and 19,488 genes	D5
Zhang et al. [21]	2013	NCBI-GEO-GSE44770	129 AD, 100 NC, and 19,488 genes	D6
Bertram et al. [24]	2007	AlzGene	22,646 genes 335 AD-associated genes	D7
Victor et al. [25]	1960	OMIM	335 AD-associated genes	D8
Greene et al. [26]	2015	GIANT	gene–gene interaction data	D9
Kerrien et al. [27]	2012	IntAct	275,145 associated genes 233 genes	D10

**Table 2:** Summary of related feature selection and classification methods for Alzheimer’s disease.

Ref.	Year	Application	Methodology		Dataset	Performance
			Feature Selection	Classification		
[12]	2016	Identification of genes related to Alzheimer’s disease with classification	- Fisher criterion - SAM - GA	SVM	D1	Accuracy = 94.55%
[13]	2016	Identification of genes related to Alzheimer’s disease with classification	Recursive feature elimination	Random Forest algorithm	D2, D3	Sensitivity = 61.0% Specificity = 70.3 % Accuracy = 65.7%
[14]	2017	Identification of genes related to Alzheimer’s disease with classification	ReliefF	- SVM - RF - ELM	D1	Sensitivity = 78.77% Specificity = 83.1 % Accuracy = 74.67%
[15]	2017	Identification of genes related to Alzheimer’s disease	- GarNet - C4.5 - QAR	- Decision Trees - Quantitative Rules - Hierarchical Cluster	D5	Best accuracy = 89.03%
[18]	2018	Identification of gene–gene interactions of Alzheimer’s disease	Sequence-based features of all genes	SVM	D7, D8, D9	Accuracy = 94% Receiver Operating Characteristic (ROC) = 84.56%
[17]	2018	Identification of gene–gene interactions of Alzheimer’s disease	22 features (statistical measurements of gene expression, correlation-based similarity measures)	Random Forest algorithm	D5, D6, D7, D10	Best results: Accuracy = 91.6% Precision = 91.6% Recall = 91.6% F-Measure = 91.6% ROC area = 96.5%
[19]	2019	Alzheimer’s disease prediction model	- Differentially expressed genes (DEG) differentially methylated positions (DMP) - “Limma package” for analysis - Integrate DEGs and DMPs by intersecting	Deep Neural Network, DNN	D5, D6	Average accuracy = 82%

Moreover, individual variability is an important consideration in the development of personalized therapies for Alzheimer’s disease [28]. As we mentioned earlier, different individuals can have varying responses to gene mutations, and this variability can play a role in disease susceptibility and progression. This is particularly relevant in complex diseases such as Alzheimer’s disease, where multiple genetic and environmental factors can contribute to disease risk and progression [28].

Our findings on the identification of genes involved in AD progression can have significant implications for personalized therapies. By identifying these genes, we can potentially develop targeted therapies that are tailored to each individual patient’s genetic makeup. For example, if a patient has a specific gene mutation that is known to contribute to AD progression, we can develop therapies that target that specific mutation [29].

In addition, our findings can help to identify potential drug targets for the treatment of Alzheimer’s disease. By identifying the genes that are most strongly associated with disease progression, we can develop drugs that target those genes and their associated pathways. This can potentially lead to more effective treatments that are tailored to each patient’s unique genetic profile [28].

Overall, our findings on the identification of genes involved in AD progression represent an important step towards the development of personalized therapies for this devastating disease. By taking into account individual variability and tailoring treatments to each patient’s unique genetic profile, we have the potential to develop more effective and targeted therapies that can slow or even halt disease progression [29].

## 4. Method

**Figure 1:**
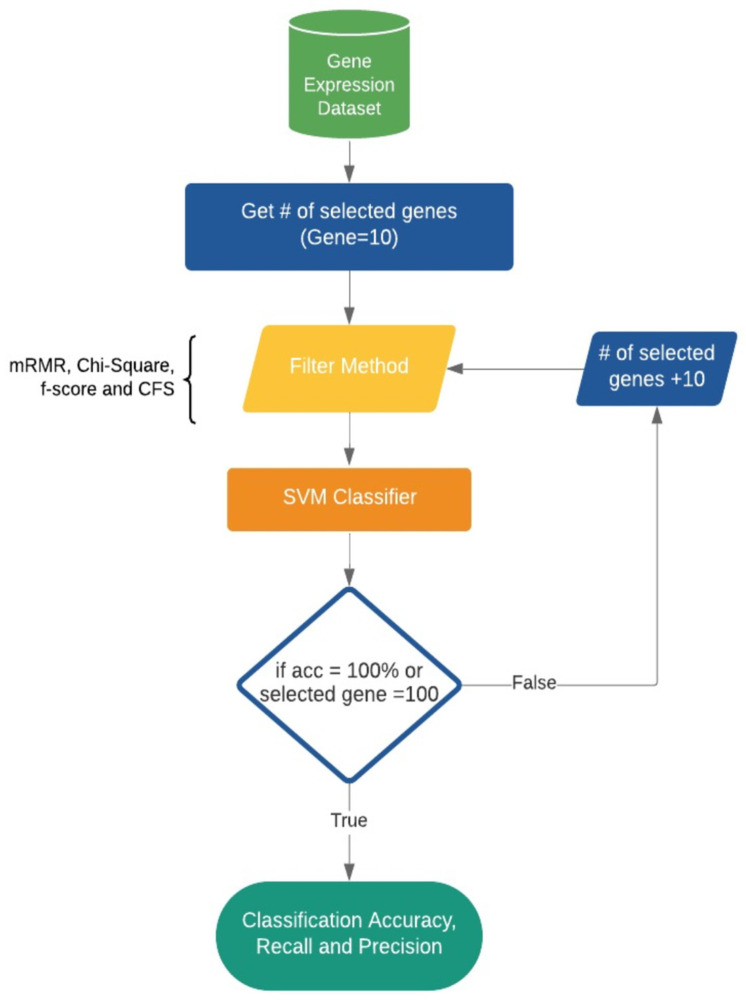
The proposed AD feature selection and classification model.

In this section, we present various methods that we used to construct our proposed model, which was developed in Python. We start by elaborating the gene expression dataset, and then we demonstrate our methods for gene selection and classification. Various types of filter gene selection methods were built into the classifier to search for an optimal subset of genes. Subsequently, the classifier identifies the genes that are highly related to AD. The below subsections further explain our wrapper model, and Figure 1 shows an overview of the model.

### 4.1. Datasets

In this study, we used a dataset published by Park et al. in [19]. The authors obtained their dataset from two integrated large-scale gene expression profiles, GSE33000 and GSE44770 [20,21]. The integrated dataset was composed of 19,488 genes, of which 257 were normal and 439 were AD samples. This dataset was normalized by z-score (https://www.ncbi.nlm.nih.gov/pmc/articles/PMC1907322/, accessed on 5 October 2021). Z-score is a transformation method that standardizes microarray datasets across a wide range of experiments. It is a useful method that facilitates data comparison and analysis. The final dataset after normalization consisted of 696 samples with 200 genes, of which 257 were normal samples and 439 were AD samples. The output format of the final dataset was .tsv format, where fields are separated by a tab. We have changed the file format to .csv (comma separated value) as it is more efficient for classification, machine learning, and data analysis in Python.

### 4.2. The Proposed Model

We built our model based on wrapper generic feature selection methods, as we evaluated all possible subsets of the genes and selected the subset that produces the best result for an SVM classifier. To select the genes for our analysis, we started by selecting a specific number of genes from the entire dataset. We began with 10 genes and evaluated their classification performance using an SVM classifier with 10-fold cross-validation. Then, we gradually increased the number of selected genes by increments of 10, resulting in the selection of 10, 20, 30, 40, 50, 60, 70, 80, 90, and 100 genes. For each set of selected genes, we again evaluated their classification performance using the same SVM classifier and 10-fold cross-validation. This approach allowed us to assess the impact of the number of selected genes on classification accuracy and identify the optimal number of genes for predicting Alzheimer’s disease based on gene expression data.

In this study, we tried both 5-fold and 10-fold cross-validation procedures and found that the 10-fold cross-validation procedure performed better in terms of predictive accuracy. The 10-fold cross-validation procedure may have performed better than the 5-fold cross-validation procedure for several reasons. First, the 10-fold cross-validation procedure generally provides a more robust estimate of model performance than 5-fold cross-validation, because it allows for a larger number of training and testing sets to be evaluated. This can help to reduce the impact of random variability in the data and provide a more stable estimate of model performance [30]. Second, the choice of the number of folds in cross-validation can depend on the size of the dataset [30]. In our study, we had a relatively large number of samples, and therefore increasing the number of folds from 5 to 10 may have allowed for a more fine-grained evaluation of the model’s performance on the data.

Eventually, we tested the predicted labels from our model by calculating the evaluation metrics and measured the performance using the following measurements.

Classification accuracy

$$Accuracy=\frac{Correct\;Predictions}{Total\;Number\;of\;Examples}$$

Precision

$$Precision=\frac{True\;Positive}{Total\;Predicted\;Positive}$$

Recall

$$Recall=\frac{True\;Positive}{Total\;Actual\;Positive}$$

These steps were repeated 11 times based on *n* to find the optimal subset of gene classification, and on the 11th time it was applied to the entire number of genes to calculate the overall accuracy of our dataset.

## 5. Results

In this section, we present the results of our experiment. First, we validated the dataset and split it into training and testing sets. We then evaluated the performance of a Support Vector Machine (SVM) classifier using 10-fold cross-validation. The SVM classifier achieved an accuracy of 0.79%.

Next, we evaluated several feature selections models with the SVM classifier and presented the genetic classification results for each filter used in our study. To analyze the measurements obtained from the feature selection models, we rounded all the values and organized them in Table 3.

From Table 3, it is clear that the minimum Redundancy Maximum Relevance (mRMR) and F-score filters outperformed all other filters, especially when the number of genes was between 20 and 80. Even though both filters produced the same results, the F-score filter was slightly better than the mRMR filter. The lowest accuracy we obtained from the mRMR filter was 0.70, while for the F-score filter it was 0.81.

The accuracy of the correlation-based feature subset selection (CFS) filter was the same for all selected numbers of genes. However, due to the long running time, we could not obtain precision and recall values.

Additionally, we trained the SVM directly with the entire dataset and obtained an accuracy of 0.79, a precision of 0.86, and a recall of 0.81.

Finally, we applied a Genetic Algorithm (GA) as a feature selection method with SVM and achieved an accuracy of 81%.

Overall, our results suggest that the use of feature selection methods, such as mRMR, F-score, and GA, can improve the accuracy of SVM classifiers in predicting AD based on gene expression data. These findings may have important implications for the development of more precise and effective diagnostic tools for AD. 

## 6. Discussion

**Figure 2:**
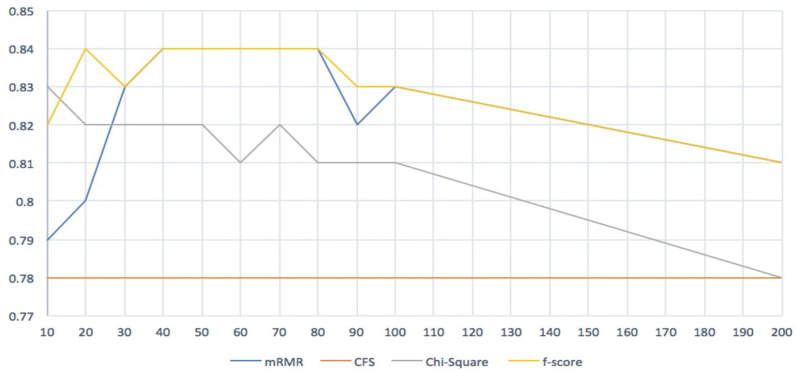
Four feature selection methods comparison.

The results of our study demonstrate the significant impact of using feature selection filter methods in enhancing the accuracy and performance of the model. Specifically, our findings suggest that the optimal number of selected genes was between 40 and 80, which resulted in the highest accuracy of 84% with both mRMR and F-score filters. Figure 2 provides a comparison of the accuracy across our model, with pilot lines emphasizing the similarity of the performance between mRMR and F-score. Notably, all classifiers produced high accuracy in the beginning, but the line of efficiency decreased as the number of selected genes increased.

In order to provide a clear understanding of the biological relevance of the selected genes, we performed several enrichment tests using Gene Set Enrichment Analysis (GSEA) [31]. Specifically, we conducted gene ontology-based enrichment tests and found that the regulation of cell death was significantly enriched from our gene set with a low *p*-value (9.36 × 10^−8^). Neuronal cell death is a frequently observed pathological feature in Alzheimer’s disease (AD), and it has been revealed that Amyloid β (Aβ), the major component of senile plaques, plays a central role in neuronal cell death. In terms of drug development, inhibiting Aβ production and aggregation is one of the representative trials [32]. Therefore, genes that can control cell death are important in AD and can also be used as diagnostic markers.

We include the results of these enrichment tests in Table 4 and provide a brief explanation of the biological relevance of the enriched GO term. In our study, we also conducted functional enrichment analysis using chemical and genetic perturbations (CGP) and applied Gene Set Enrichment Analysis (GSEA) to assess the functional relevance of our identified features to Alzheimer’s disease (AD). The results of the analysis showed that 40 genes significantly overlapped with groups of AD-related downregulated genes, as indicated in Table 5.

This finding suggests that the feature we extracted is closely associated with AD-related functions or pathways. The functional enrichment test using CGP provides an important tool for identifying the underlying biological mechanisms of complex diseases like AD. By identifying the gene sets that are significantly associated with AD-related functions or pathways, we can gain insights into the molecular mechanisms underlying the disease and identify potential therapeutic targets. The results of the functional enrichment tests suggest that the feature we extracted is significantly associated with AD-related functions or pathways.

Table 6 presents the best performances and comparison between four filter feature selection methods studied in this research, along with the SVM classifier classification accuracy and the number of selected genes. The results show that the mRMR and F-score filters achieved the highest classification accuracy of 84%, with 40 and 20 genes selected, respectively. The Chi-Square filter achieved an accuracy of 83% with 10 genes selected, while the CFS filter had a lower accuracy of 78% with only 10 genes selected. These findings suggest that the mRMR and F-score filters are effective feature selection methods for predicting Alzheimer’s disease based on gene expression data, outperforming the Chi-Square and CFS filters. Additionally, the results show that the number of selected genes has a significant impact on classification accuracy, with a larger number of genes generally resulting in higher accuracy.

Our study’s results are particularly noteworthy because they outperformed a deep learning model, Deep Neural Network (DNN), proposed by Park et al. [19]. Specifically, our mRMR and F-score filters achieved an accuracy of 84%, while the DNN model achieved an accuracy of 82% on the same gene expression dataset.

The results of our study have important implications for the development of more accurate and effective diagnostic tools for AD. By identifying the most relevant genes associated with the disease, we can develop more targeted treatments and therapies that may slow or even prevent the onset of AD.

However, it is important to note that our study had some limitations. For example, we used a single gene expression dataset, and future research should examine the generalizability of our findings to other datasets. Additionally, our study did not examine the potential interactions between genes, and future research should explore this area to further enhance the accuracy of AD prediction models.

Overall, our study’s findings highlight the importance of feature selection filter methods in improving the accuracy and performance of AD prediction models and provide a foundation for future research in this area.

## 7. Conclusions

In this study, we evaluated several feature selection methods and classifiers for predicting Alzheimer’s disease (AD) based on gene expression data. Our analysis of the dataset modified by Park et al. [19] revealed that the mRMR and F-score filters achieved the highest classification accuracy of 84% with a gene number of 20–40. Our findings also showed that the mRMR and F-score filters outperformed the GA, Chi-Square Test, and CFS methods in terms of classification accuracy. These results suggest that the mRMR and F-score filters are effective feature selection methods for predicting AD based on gene expression data. Our findings could have important implications for the development of more accurate diagnostic tools and potential therapeutic targets for AD.

Our findings demonstrate the importance of feature selection methods in improving the accuracy of AD prediction models, and our study provides a foundation for future research in this area. Specifically, future studies should examine the generalizability of our findings to different datasets and evaluate the effectiveness of different models on these datasets. In future work, we plan to conduct additional analyses to assess the consistency of the selected genes across different folds. Our aim is to compare the genes selected in each fold and evaluate the degree of overlap between them. This will allow us to identify which genes are consistently selected across different folds and which genes may exhibit greater variability. Furthermore, future research should explore the potential of hybrid approaches that combine filter and wrapper models for feature selection in AD prediction. Overall, our study’s findings have important implications for the development of more accurate and effective diagnostic tools for AD. By identifying the most relevant genes associated with the disease, we can develop more targeted treatments and therapies that may slow or even prevent the onset of AD.

## Figures and Tables

**Table 3:** The classification results for filter feature selection method under study with SVM classifier using different number of genes.

	SVM									
# of Genes	mRMR			CFS	Chi-Square			F-Score		
	Accuracy	Precision	Recall	Accuracy	Accuracy	Precision	Recall	Accuracy	Precision	Recall
10	0.79	0.80	0.88	0.78	0.83	0.83	0.93	0.82	0.84	0.89
20	0.80	0.82	0.88	0.78	0.82	0.82	0.92	0.84	0.85	0.90
30	0.83	0.84	0.89	0.78	0.82	0.83	0.91	0.83	0.85	0.90
40	0.84	0.86	0.90	0.78	0.82	0.84	0.90	0.84	0.85	0.90
50	0.84	0.85	0.90	0.78	0.82	0.84	0.89	0.84	0.86	0.89
60	0.84	0.85	0.90	0.78	0.81	0.83	0.89	0.84	0.86	0.90
70	0.84	0.86	0.89	0.78	0.82	0.85	0.88	0.84	0.86	0.89
80	0.84	0.85	0.90	0.78	0.81	0.84	0.87	0.84	0.86	0.89
90	0.82	0.85	0.88	0.78	0.81	0.84	0.86	0.83	0.86	0.87
100	0.83	0.86	0.88	0.78	0.81	0.85	0.85	0.83	0.86	0.87
All	0.81	0.85	0.84	0.78	0.78	0.84	0.81	0.81	0.85	0.85

**Table 4:** Result of functional enrichment test with gene ontology and KEGG pathway by using GSEA.

Category	GO Term or KEGG Pathway	*p*-Value
Gene ontology biological process	Regulation of cell death	9.36 × 10^−8^

**Table 5:** The outcome of the functional enrichment analysis using chemical and genetic perturbations (CGP) in Gene Set Enrichment Analysis (GSEA) revealed results that are related to Alzheimer’s disease (AD).

GCP	Description	*p*-Value
Blalock Alzheimer’s disease	Genes downregulated in brain from patients with Alzheimer’s disease	1.87 × 10^−7^

**Table 6:** Best performance comparison between filter feature selection methods under study with SVM classifier classification accuracy and number of selected genes.

Methods	Classification Accuracy	# of Genes
mRMR + SVM	84%	40
CFS + SVM	78%	10
Chi-Square + SVM	83%	10
F-score + SVM	84%	20

## Data Availability

In this study, we used a dataset published by Park et al. in [19]. The authors obtained their dataset from two integrated large-scale gene expression profiles, GSE33000 and GSE44770 [20,21]. The integrated dataset was composed of 19,488 genes, of which 257 were normal and 439 were AD samples. This dataset was normalized by z-score (https://www.ncbi.nlm.nih.gov/pmc/articles/PMC1907322/, accessed on 5 September 2019).
